# Deep Brain Stimulation in Treatment-Resistant Psychiatric Disorders: Efficacy, Safety, and Future Directions

**DOI:** 10.3390/brainsci15111244

**Published:** 2025-11-20

**Authors:** Mohsen Khosravi

**Affiliations:** 1 Department of Psychiatry, School of Medicine, Zahedan University of Medical Sciences, Zahedan 9813913777, Iran; dr_khosravi2016@yahoo.com or m.khosravi@zaums.ac.ir; Tel.: +98-5433522636; Fax: +98-5433518352; 2 Health Promotion Research Center, Zahedan University of Medical Sciences, Zahedan 9816743463, Iran; 3 Community Nursing Research Center, Zahedan University of Medical Sciences, Zahedan 9816743463, Iran

**Keywords:** deep brain stimulation, treatment-resistant, psychiatric disorders, safety, efficacy

## Abstract

Treatment-resistant psychiatric disorders represent a major clinical challenge, with a significant proportion of patients remaining refractory to conventional pharmacological and psychotherapeutic interventions. Deep brain stimulation (DBS), a neurosurgical technique delivering targeted electrical impulses to specific brain regions, has emerged as a promising intervention across a spectrum of refractory psychiatric conditions. This comprehensive narrative review synthesizes current evidence on the efficacy, safety, and practical considerations of DBS for treatment-resistant major depressive disorder, obsessive–compulsive disorder, bipolar disorder, schizophrenia, addictions, Tourette’s syndrome, anorexia nervosa, post-traumatic stress disorder, and refractory aggression in autism spectrum disorder with severe intellectual disability. Across most conditions, DBS demonstrates clinically meaningful symptom reductions, with response and remission rates in depression and obsessive–compulsive disorder approaching 48% and 35%, respectively. For Tourette’s syndrome and refractory aggression in autism, over two-thirds of patients’ experience > 50% symptom reduction. Preliminary data in bipolar disorder, schizophrenia, addictions, and anorexia nervosa are encouraging but limited by small sample sizes and methodological heterogeneity. Safety profiles are generally acceptable, with the majority of adverse events being device- or procedure-related; psychiatric adverse effects and rare serious complications underscore the importance of careful patient selection and monitoring. However, the literature is constrained by inconsistent study designs, a paucity of randomized controlled trials, heterogeneity in DBS targets and stimulation parameters, and limited long-term and quality-of-life outcomes. Optimization of anatomical targeting, stimulation protocols, and patient selection criteria remains an ongoing challenge. Future directions require larger, rigorously controlled trials with standardized outcome measures, integration of neurobiological biomarkers, and multidisciplinary collaboration. In summary, while DBS offers transformative potential for select cases of refractory psychiatric illness, its application must be guided by scientific rigor, ethical prudence, and individualized patient-centered care.

## 1. Introduction

Psychiatric disorders are a significant global health issue and are among the main causes of disability worldwide [1]. Despite advances in pharmacological and psychotherapeutic interventions, a substantial proportion of patients (20–60%) remain unresponsive to conventional treatments—a phenomenon commonly referred to as treatment resistance [2]. Treatment-resistant psychiatric disorders present profound challenges to individuals, families, and healthcare systems, often resulting in chronic suffering, functional impairment, increased healthcare utilization, and an elevated risk of suicide [2,3]. This clinical impasse has galvanized research into novel therapeutic strategies capable of providing relief for patients who have exhausted standard options.

In recent years, the field of neuropsychiatry has witnessed a paradigm shift with the introduction and development of neuromodulation techniques. Among these, deep brain stimulation (DBS)—a neurosurgical intervention involving the delivery of electrical impulses to specific brain regions—has emerged as a promising candidate for the management of treatment-resistant psychiatric disorders. Originally developed for the treatment of movement disorders such as Parkinson’s disease and essential tremor, DBS has demonstrated remarkable efficacy and safety in these populations, spurring interest in its application to psychiatric conditions [4]. Recent advances in the understanding of dysfunctional neurocircuitry underlying psychiatric illnesses have provided a mechanistic basis for neuromodulation approaches such as DBS, enabling more precise targeting of brain networks implicated in symptom generation [5,6,7]. The potential of DBS to modulate abnormal neural circuits in treatment-resistant psychiatric disorders offers hope to patients who have not benefited from traditional therapies [8]. Preliminary studies and case reports have provided encouraging evidence of symptom improvement in select populations with treatment-resistant psychiatric disorders. However, the field remains in its infancy, with heterogeneous clinical outcomes, limited sample sizes, and variability in patient selection criteria and stimulation parameters [9]. Moreover, ethical considerations, long-term safety, and the mechanisms underlying clinical response require further elucidation [10,11]. Given the invasive and resource-intensive nature of DBS, ethical and translational aspects—such as patient consent, equitable access, and implications for psychiatric care—are critically important and must be explicitly considered when assessing its clinical utility. A rigorous appraisal of its efficacy and safety profile is essential to inform clinical decision-making and guide future research directions.

This narrative review aims to provide a comprehensive and up-to-date synthesis of the efficacy and safety of DBS for treatment-resistant psychiatric disorders. Our objectives are as follows: (i) Evaluate clinical outcomes and adverse effects associated with DBS in these populations; (ii) Examine factors influencing therapeutic response, including patient and target selection, as well as stimulation parameters; (iii) Identify current gaps in the literature and propose priorities for future research; and (iv) Explicitly address ethical and translational considerations relevant to the adoption of DBS in psychiatric practice. In contrast to previous meta-analyses or systematic reviews, this review places special emphasis on recent clinical trials, the integration of neurocircuitry findings with therapeutic innovation, and a critical appraisal of challenges and advances in the field, thereby offering novel insights into the evolving landscape of DBS for psychiatric disorders. By critically evaluating the existing body of evidence, this review aims to clarify the therapeutic potential of DBS in psychiatry and to inform clinicians, researchers, and policymakers about its current status and future prospects in the management of refractory psychiatric illnesses.

## 2. Methods

### 2.1. Search Strategy and Study Selection

**Figure 1:**
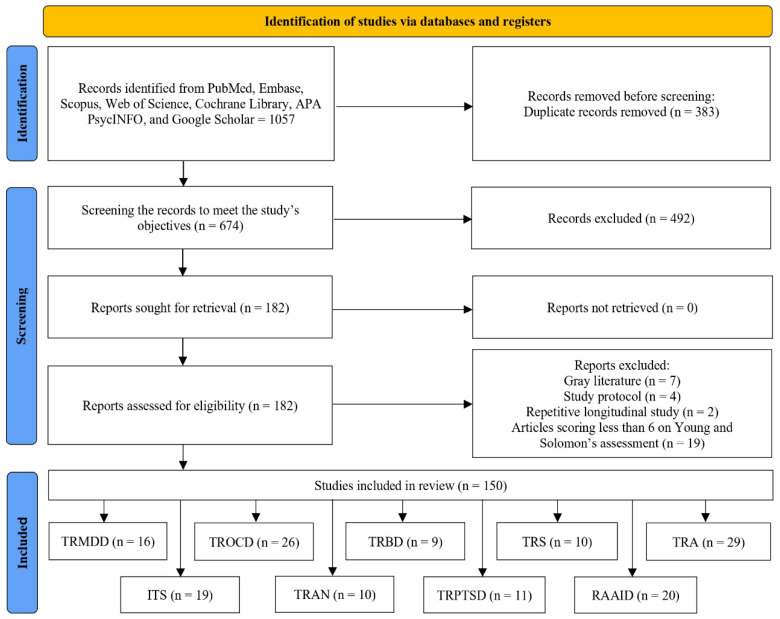
PRISMA flow diagram. Note. TRMDD—treatment-resistant major depressive disorder; TROCD—treatment-resistant obsessive–compulsive disorder; TRBD—treatment-resistant bipolar disorder; TRS—treatment-resistant schizophrenia; TRA—treatment-refractory addictions; ITS—intractable Tourette’s syndrome; TRAN—treatment-refractory anorexia nervosa; TRPTSD—treatment-refractory post-traumatic stress disorder; RAAID—refractory aggression in autistic children with severe intellectual disability.

A comprehensive search of electronic databases (e.g., PubMed, Embase, Scopus, Web of Science, Cochrane Library, APA PsycINFO, and Google Scholar) was performed using combinations of relevant keywords, including “deep brain stimulation”, “DBS”, “brain neurostimulation”, “neuromodulation”, “treatment-resistant”, and “psychiatric disorders”. The search was limited to articles published in English up to June 2025. Specifically, the search strategy used combinations such as (“deep brain stimulation” OR “DBS” OR “brain neurostimulation” OR “neuromodulation”) AND (“treatment-resistant”) AND (“psychiatric disorders”). The total number of articles retrieved from all databases was 1057, and after screening titles and abstracts, 150 studies were included for full-text review (see Figure 1 for detailed search strategy and results). Additional studies were identified by screening the reference lists of included articles. Studies were eligible for inclusion if they (i) reported on human subjects with treatment-resistant psychiatric disorders, (ii) described the application of DBS as an intervention, and (iii) provided clinical outcomes. Observational studies, randomized controlled trials (RCTs), case reports, case series, and narrative and systematic reviews were included, whereas editorials and conference abstracts were excluded.

### 2.2. Data Extraction

For each included study, relevant data were extracted, including electrode placement, stimulation parameters, length of follow-up (months), primary outcome measure(s), mean score improvement (%), responders, and adverse events. Two reviewers independently reviewed and extracted data, with disagreements resolved by consensus with a third reviewer. Although the decision to include both narrative and systematic reviews within the synthesis is pragmatic, it introduces potential overlap between sources; this limitation was explicitly considered and discussed in the interpretation of the results.

### 2.3. Appraisal of Study Quality

Each selected article was evaluated using 10 assessment questions from Young and Solomon [12]. For each question, a score of 1 was given if the criterion was met, and 0 if it was not met or unclear. The total score for each article determined its quality: poor (3 or less), fair (4 or 5), good (6 to 8), or high quality (9 or 10). This systematic scoring method ensured consistent and objective appraisement of the articles included in the review, enabling clear comparison of their methodological quality. The appraisal was independently performed by two reviewers, with discrepancies resolved through discussion with a third reviewer. At the end, only articles that obtained a score of 6 or higher were considered in this narrative review.

### 2.4. Synthesis of Evidence

Data from the included studies were synthesized narratively, following McLure’s technique [13], to provide a comprehensive overview of the efficacy, influencing factors, and safety of DBS in treatment-resistant psychiatric disorders. Particular attention was paid to study design, DBS targets, stimulation parameters, outcomes, and adverse events. Gaps in the literature and areas for future research were also highlighted.

## 3. Results

The initial results of our review are summarized in two tables. Table 1 provides a comparative overview of the efficacy of DBS across various treatment-resistant psychiatric disorders, highlighting differences in therapeutic outcomes. Table 2 presents a statistical summary of the most common side effects associated with DBS. In the following sections, we discuss in detail the application of DBS for each psychiatric disorder and examine the clinical outcomes.

### 3.1. Treatment-Resistant Major Depressive Disorder

DBS has emerged as a promising intervention for treatment-resistant depression, with a growing body of evidence evaluating its efficacy and safety. Across studies, DBS demonstrated a substantial reduction in depressive symptomatology, as measured by standardized rating scales such as the Montgomery–Åsberg Depression Rating Scale and the Hamilton Depression Rating Scale, with an observed average improvement of approximately 47% at intermediate to long-term follow-up (21 months on average). The model-based estimation indicated that the time required to reach a 50% improvement threshold in responders was about 23 months, underscoring the gradual but meaningful trajectory of symptomatic relief associated with the intervention. The impact of the stimulation target was also explored, revealing a trend toward differential efficacy across neural targets. In some analyses, the medial forebrain bundle and ventral capsule/ventral striatum appeared to show greater improvements than the nucleus accumbens, subgenual cingulate gyrus/cortex, bed nucleus of the stria terminalis, thalamic peduncles, lateral habenula, and the ventral part of the anterior limb of the internal capsule. However, these target-specific effects did not reach statistical significance, likely due to limited sample sizes and heterogeneity in stimulation parameters, precluding the formulation of definitive conclusions regarding the superiority of any particular anatomical target. Target selection accounted for a significant proportion (up to 40%) of cross-study variability, highlighting the necessity for further investigation with more granular data [14,15,16].

Our review indicated that efficacy optimization algorithms typically begin by increasing amplitude, followed by adjusting electrode contacts or pulse width. High-frequency stimulation (>100 Hz) was consistently used across brain targets. Three primary parameter combinations were identified: (i) short pulse width (60–90 μs) with low amplitude (0–4 V), (ii) short pulse width with high amplitude (5–10 V), and (iii) long pulse width (120–450 μs) with low amplitude. However, no significant difference in mean stimulation parameters was observed between responders and non-responders, highlighting the influence of factors beyond stimulation settings in determining clinical outcomes [17].

Study design emerged as a key determinant of observed efficacy. Open-label trials consistently reported greater improvements compared to RCTs, with an absolute difference exceeding 20% in favor of open-label designs [18]. This discrepancy is in line with prior qualitative syntheses and may reflect both methodological and population differences; open-label studies tend to enroll smaller, less severely affected cohorts and may be more susceptible to expectancy effects, whereas RCTs often involve larger, more rigorously selected populations, frequently conducted in academic centers or with industry collaboration. Such context may introduce additional challenges, including more refractory disease and stricter outcome assessments, potentially attenuating observed treatment effects [19]. The anticipated results of ongoing large-scale RCTs, such as the TRANSCEND trial, are expected to further clarify these patterns and provide a more robust comparative framework for interpreting efficacy outcomes [20]. However, patient demographic and clinical characteristics—such as sex, age at onset, and illness duration—did not significantly influence long-term treatment outcomes in analyses that incorporated individual patient-level data. The limited availability of such data necessitates cautious interpretation, and further studies with larger and more diverse samples are required to definitively characterize potential moderators of response. Similarly, the duration of follow-up did not appear to impact the extent of improvement, which is consistent with the modeled time course suggesting that the greatest incremental benefit occurs within the first 12 months, followed by a plateau. This pattern aligns with the minimum study duration required for inclusion in this review [14,15,16].

A critical finding of the present synthesis pertains to the comparison of active versus sham stimulation in RCTs. No statistically significant difference in symptomatic improvement was detected between these arms, mirroring the primary outcomes of several individual studies included in the analysis [21,22,23]. This lack of distinction may be attributable to considerable variance within the sham groups, compounded by the restricted sample size and brief duration of sham exposure. Several mechanisms could underlie the apparent efficacy of sham stimulation, including the so-called “implant effect”—temporary improvement following electrode placement, possibly linked to microlesioning—cognitive placebo effects, and inherent fluctuations in depressive symptom trajectories [24,25]. The pronounced effects observed in open-label trials and the notable response rates in sham arms complicate efforts to disentangle the specific therapeutic benefits of stimulation from those of indirect or nonspecific factors, such as study design or patient expectations. While a prior meta-analysis reported superiority of active over sham stimulation in double-blind crossover designs, such findings were restricted to single time points at the end of each crossover phase [19]. In the present synthesis, active stimulation displayed greater efficacy at selected time intervals (notably at 3 and 6 months), but the aggregation of data from heterogeneous time points led to convergence of the overall time courses between active and sham groups. This highlights the importance of standardized assessment timelines and longer sham periods to optimize the sensitivity of future comparative analyses.

Long-term response and remission rates were also evaluated, with findings indicating rates of 48% and 35%, respectively, paralleling those reported in previous investigations. The model-based estimate for achieving a 50% responder rate mirrored the improvement metric, occurring at approximately 21 months. Consistent with the overall improvement findings, the time courses for response rates in active and sham arms were largely overlapping [14,15,16,17,18,19].

Safety outcomes were systematically reported across the included studies. Serious adverse events encompassed device-related complications, infections, neurological sequelae, and suicidality. Device-related complications, including lead removal or replacement due to impaired stimulation delivery or wound issues, occurred in a minority of patients and were generally manageable with secondary interventions. Device battery depletion was associated with relapse of depressive symptoms in a small subset, with symptomatic improvement following battery replacement [14]. Infectious complications were relatively infrequent, with postoperative infections constituting a minor proportion of adverse events. Seizure incidence was exceedingly rare. Notably, rates of suicidal ideation and suicide attempts, as well as completed suicides, were systematically tracked. The observed suicide rate (0.87/100 person-years) was comparable to those reported in previous neuromodulation studies and large-cohort observational databases [26,27,28]. The slightly elevated rates in the present synthesis are plausibly attributable to the inclusion of more severely affected treatment-resistant depression cohorts, characterized by stringent eligibility criteria, prolonged illness duration, and documented failure of multiple treatment modalities. Importantly, causal attribution between DBS and suicidal behavior remains contentious, with deaths by suicide variably associated with nonresponse, impulsivity, or occurring outside the defined study windows. Collectively, the available evidence does not implicate DBS as a direct contributor to increased suicide risk in treatment-resistant depression populations [26,29].

In addition to these results, several methodological limitations were identified. Many studies lacked comprehensive reporting of patient-level data, precluding more nuanced subgroup and network analyses. The inability to account for detailed stimulation settings at the individual level represents a further challenge, particularly as advances in personalized targeting are likely to increase parameter heterogeneity and complicate cross-study comparisons. Moreover, the need for patient-specific optimization and the potential for unintentional unblinding during discontinuation phases complicate the interpretation of placebo-controlled periods. Longer-term sham control periods with frequent outcome assessments would enhance the resolution of time course analyses, but they are challenging to implement due to difficulties in maintaining blinding over extended durations.

### 3.2. Treatment-Resistant Obsessive–Compulsive Disorder

The literature on surgical and neuromodulatory interventions for refractory obsessive–compulsive disorder has grown considerably over the past two decades, with a focus on individuals presenting with severe to extreme symptoms that are unresponsive to multiple evidence-based therapies. Across the included body of research, the populations studied have been relatively homogeneous in terms of clinical severity and chronicity. Participants were consistently adults, with mean ages in the early forties, and universally met stringent criteria for treatment resistance, including failed trials of both first-line pharmacotherapies—commonly selective serotonin reuptake inhibitors and clomipramine—as well as adjunctive medication regimens and extensive expert-delivered exposure and response prevention therapy. At baseline, the severity of obsessive–compulsive disorder was high, as indicated by Yale-Brown Obsessive Compulsive Scale scores averaging above 30, a threshold corresponding to severe disease [30,31,32,33,34,35,36,37,38,39,40,41,42,43,44,45,46,47,48,49,50,51].

Durations of illness were notably prolonged, with the majority of studies mandating a minimum of five years of unremitted symptoms prior to consideration for surgical intervention, reflecting a consensus on the use of these procedures in only the most refractory cases. Some investigations required even longer periods of chronicity or failed to specify minimum durations, introducing some heterogeneity in this respect [32,51,52]. Across the literature, psychiatric comorbidities were frequent, with major depressive disorder present in more than half of cases, and additional anxiety and personality disorders reported with lower but substantial prevalence, further underscoring the complexity of this clinical population [39,41,42,45,47,48].

A diversity of neuroanatomical targets for DBS and related interventions has been explored, reflecting evolving hypotheses regarding the neural circuitry underpinning obsessive–compulsive disorder. The ventral capsule/ventral striatum and the nucleus accumbens have emerged as the most frequently investigated loci, with the anterior limb of the internal capsule, bed nucleus of the stria terminalis, and subthalamic nucleus also receiving substantial attention [30,31,32,34,36,39,42,43,44,46,49,50,51]. Some studies have evaluated combined or less conventional targets, including the inferior thalamic peduncle, caudate nucleus, and various thalamic nuclei, reflecting a broadening of neurosurgical strategies as understanding of obsessive–compulsive disorder pathophysiology deepens [36,37,40,41,45,47,49,52]. Stimulation was mostly bilateral, starting 6–8 weeks post-surgery, with DBS settings of 4–6 V, 60–120 μs pulse width, and 130–180 Hz frequency [53].

Safety profiles were a central focus across the literature, with serious adverse events systematically captured by the majority of studies. The most frequently observed device-related complications included lead damage or malposition, and postoperative infections, some of which necessitated explantation or generator replacement [31,45]. Seizures occurred at a rate consistent with other intracranial procedures, and isolated incidents of intracranial hemorrhage were described, occasionally resulting in lasting neurological deficits such as dysarthria or finger palsy [51]. Psychological complications were also noted, including cases in which the neurostimulation device itself became the focus of new obsessive-compulsive symptoms, exemplifying the complex interplay between device intervention and the psychopathology of obsessive–compulsive disorder [45]. Suicidality was reported both as an adverse event and as a background risk, with rare instances of suicide attempts and a completed suicide documented, consistent with the high baseline risk in this patient population. Overall, while a significant minority of patients experienced serious adverse events, the majority of these events were manageable and comparable to those reported in other neurosurgical series.

Across the synthesized literature, robust and clinically meaningful improvements in obsessive–compulsive disorder severity were observed following intervention. Meta-analytic pooling of Yale-Brown Obsessive Compulsive Scale outcome data revealed a mean reduction in symptom severity exceeding 14 points at final follow-up, corresponding to a substantial proportionate decrease relative to baseline [30,31,32,33,34,35,36,37,38,39,40,41,42,43,44,45,46,49,50,51]. When analyzed in terms of percentage change, the mean reduction approached 50%, with responder rates—defined by standard criteria as a reduction of the Yale-Brown Obsessive Compulsive Scale by at least 35%—consistently in the range of two-thirds of treated individuals. These improvements were statistically significant across the pooled analyses and were observed across different target sites, though direct comparisons of efficacy between targets were limited by the heterogeneity of studies and lack of adequately powered head-to-head trials. Reductions in comorbid depressive and anxiety symptoms were also frequently reported, though the magnitude of these effects was generally smaller and more variable than for primary obsessive–compulsive disorder symptoms. Functional gains, as measured by Global Assessment of Functioning and other global measures, were less consistently reported, but when available, suggested improvements in daily functioning in parallel with symptom reduction [54].

The accumulated evidence supports the efficacy of neurosurgical and DBS interventions for the most severely affected and treatment-refractory obsessive–compulsive disorder patients. These interventions yield large and durable reductions in symptom severity for most individuals, with a safety profile that, although not trivial, is acceptable given the severity and chronicity of the target population [54,55]. Methodological limitations, particularly in the nonrandomized literature, and the diversity of stimulation targets and protocols, preclude firm conclusions regarding optimal approaches. Nonetheless, the convergence of findings across a range of study designs and clinical settings provides a compelling argument for the continued development and refinement of neuromodulatory treatments in severe obsessive–compulsive disorder.

### 3.3. Treatment-Resistant Bipolar Disorder

The evidence base for DBS in treatment-resistant bipolar disorder is currently restricted, with few studies directly examining its efficacy in this population [56]. The available data, though sparse, provide preliminary insights. In the largest study to date, individuals diagnosed with bipolar disorder type II demonstrated a decrease in depressive symptoms that was comparable to the improvement observed in individuals with major depressive disorder [57]. This finding suggests potential parity in the antidepressant effects of DBS across these diagnostic categories, supporting its consideration as a therapeutic modality in bipolar depression. Further support for the efficacy of DBS in treatment-resistant bipolar disorder comes from case reports, wherein individuals with longstanding, treatment-resistant bipolar depression experienced significant symptomatic improvement following DBS. Notably, one such report detailed a patient who maintained a robust antidepressant response throughout a nine-month treatment period [58]. While these accounts are inherently limited by their observational and anecdotal nature, they nevertheless underscore the potential of DBS to induce and sustain remission in otherwise refractory cases.

To date, no controlled trials have systematically evaluated the effectiveness of DBS across the full spectrum of bipolar disorder presentations, and there is a conspicuous lack of published data addressing its impact on other bipolar disorder states beyond depression. These gaps highlight the nascent stage of research in this domain and the pressing need for rigorous, large-scale investigations to establish efficacy, delineate optimal patient selection criteria, and inform clinical protocols [56,57,58]. An additional open-label pilot study, which included participants with both treatment-resistant depression and treatment-resistant bipolar disorder, reported favorable outcomes [59]. The degree of symptomatic improvement in depressive dimensions was noted to be similar irrespective of diagnostic subgroup, further substantiating the antidepressant potential of DBS in affective disorders. These findings, while promising, require replication and extension through RCTs employing robust methodological designs to mitigate bias and confirm treatment effects.

The cognitive sequelae of DBS, particularly within the context of bipolar disorders, remain underexplored. Despite the proliferation of RCTs and observational studies assessing DBS in other neuropsychiatric populations, no studies have directly investigated its impact on cognitive functioning in individuals with bipolar disorders. This represents a substantial knowledge gap, as cognitive impairment constitutes a clinically significant and functionally disabling aspect of bipolar illness and may be susceptible to neuromodulatory interventions. In addition, to date, there are no published systematic investigations focused on the side effect profile or safety outcomes of DBS in individuals with treatment-resistant bipolar disorder, underscoring an urgent need for targeted safety studies [60]. Nevertheless, isolated case reports and clinical observations have documented instances of treatment-emergent (hypo)manic symptoms following DBS in patients with Parkinson’s disease, major depressive disorder, or obsessive–compulsive disorder [61,62,63]. These affective switches, while relatively infrequent, are of particular clinical concern in the bipolar population, where mood instability represents a core feature of the disorder. A review examining the use of DBS in bipolar disorder identified a case in which a patient experienced two discrete episodes of hypomania during the course of treatment [56]. Importantly, these episodes were resolved with titration and adjustment of stimulation parameters, indicating that careful monitoring and individualized programming may mitigate the risk of mood destabilization [60]. In contrast, the largest study to date involving bipolar disorder patients treated with DBS found no evidence of treatment-emergent affective switches, suggesting that, with vigilant management, the risk may be acceptably low [56]. These findings, while reassuring, require confirmation in larger cohorts and controlled settings to establish the generalizability and reproducibility of safety outcomes.

In summary, the extant literature on DBS for treatment-resistant bipolar disorders is characterized by a limited but growing evidence base. Preliminary findings suggest that DBS may confer significant antidepressant effects in individuals with treatment-resistant bipolar depression, with a safety profile that appears manageable with appropriate monitoring and parameter adjustment [56,57,58,61,64]. However, the lack of RCTs, the scarcity of data on cognitive and long-term safety outcomes, and the potential for affective destabilization highlight the imperative for further research. Future studies employing rigorous experimental designs are required to delineate the role of DBS in the therapeutic armamentarium for bipolar disorders, define optimal patient selection, and establish standardized protocols for clinical use.

### 3.4. Treatment-Resistant Schizophrenia

Emerging evidence from a limited but growing body of clinical research suggests that DBS may offer therapeutic potential for individuals with treatment-resistant schizophrenia. The studies conducted to date, though small in scale and methodologically diverse, have explored the use of DBS across a variety of neuroanatomical targets implicated in the pathophysiology of schizophrenia, yielding nuanced insights into both the efficacy and safety profile of this intervention. Across the published case reports, case series, and a pivotal pilot randomized crossover trial, several brain regions have been targeted for DBS in schizophrenia, including the subgenual anterior cingulate cortex, nucleus accumbens, habenula, and substantia nigra pars reticulata [65,66,67,68,69]. The rationale for targeting these regions is strongly grounded in the neurobiological mechanisms underlying schizophrenia. For example, the subgenual anterior cingulate cortex is a central node within the default mode network, which is often dysregulated in this disorder, potentially contributing to disturbed cognitive and emotional processing. Targeting the nucleus accumbens aligns with prevailing models of dopaminergic dysfunction, as this region is critical for mediating dopamine release and is thought to be influenced by aberrant hippocampal input. The substantia nigra, a central hub in the dopamine system, further reflects the centrality of dopaminergic imbalance in schizophrenia symptomatology, while the habenula’s regulatory influence on midbrain dopaminergic centers positions it as another promising target [70,71,72,73,74]. Clinical outcomes following DBS in these brain regions have demonstrated variable, but generally positive, effects on both positive and negative symptoms of schizophrenia. The majority of patients across these studies showed improvement in standardized clinical outcomes, such as the Positive and Negative Syndrome Scale and the Brief Psychiatric Rating Scale, following DBS during a 10–12 months stimulation period. While the degree of improvement varied, several patients reached the threshold of clinical response, commonly defined as a reduction of at least 25% in total Positive and Negative Syndrome Scale or Brief Psychiatric Rating Scale scores [65,66,67,68,69]. Notably, the beneficial effects of DBS were not uniformly distributed across all patients or all symptom domains. For instance, some individuals experienced significant reductions in positive symptoms—such as hallucinations and delusions—while the impact on negative symptoms, including affective flattening and social withdrawal, was less consistent [65].

Detailed longitudinal follow-up, as seen in the three-year outcomes from the randomized trial, provides further insight into the durability and pattern of response [69]. Among the patients who achieved an initial significant improvement, continued stimulation was associated with sustained symptom reduction over extended periods. However, withdrawal or interruption of active stimulation consistently led to a worsening of symptoms—sometimes acutely—underscoring both the therapeutic effect of DBS and the potential for symptom relapse upon cessation. In crossover trial phases, patients who transitioned from active to sham or no stimulation experienced marked exacerbations of both positive and negative symptoms, with some unable to complete the phase due to symptom deterioration. This pattern reinforces the hypothesis that DBS exerts a direct modulatory effect on neural circuits that underlie the core pathology of schizophrenia. Nevertheless, the response to DBS appears to be heterogeneous. While some patients achieved robust and sustained clinical improvement, others had only partial responses, and in rare cases, clinical deterioration was observed. For example, one patient demonstrated initial decreases in Positive and Negative Syndrome Scale scores but ultimately relapsed into a psychotic episode, resulting in withdrawal from the study. In another case, DBS targeting the subgenual anterior cingulate cortex or nucleus accumbens led to increased negative symptoms despite overall reductions in positive symptomatology. Cognitive outcomes, as assessed by neuropsychological testing, were mixed; certain patients showed improvements in domains such as verbal fluency, while others experienced declines in learning and memory performance, suggesting that DBS may exert complex and region-dependent effects on cognitive function [69].

The parameters for stimulation and amplitude settings for DBS in treatment-resistant schizophrenia were as follows: Stimulation began 48–72 h post-surgery, using unilateral left-sided stimulation (contact as anode, case as cathode). The initial settings were an amplitude of 2.5 V, a pulse width of 60 μs, and a frequency of 130 Hz [68].

The safety profile of DBS in this population has also been characterized in these early studies. Adverse events occurred but were generally manageable with clinical intervention and did not result in permanent deficits. The most significant adverse events included a postoperative hemorrhage and device infection necessitating hardware removal, seizures controlled with antiepileptics, and mood instability sometimes associated with discontinuation of concurrent antipsychotic therapy. Transient adverse effects were also reported, such as akathisia responsive to adjustment of stimulation parameters, electrical sensations, perioperative confusion, and increased appetite leading to a weight gain of 33 pounds (15 kg). Importantly, the majority of serious adverse events were concentrated in a single patient, while other side effects were self-limiting or resolved with changes in stimulation modality or concomitant medication [65,66,67,68,69].

Taken together, the current body of clinical research on DBS for treatment-resistant schizophrenia suggests a cautiously optimistic outlook. The data indicate that DBS, when targeted to relevant brain circuits, can yield clinically meaningful improvements in a subset of severely affected patients who have not responded to standard pharmacological or psychosocial interventions. The observation that symptom exacerbation reliably follows the withdrawal of stimulation further strengthens the case for a direct therapeutic mechanism [65,66,67,68,69]. Nevertheless, the overall heterogeneity in response, the persistence or worsening of negative symptoms in certain cases, and the occurrence of both transient and serious adverse events underscore the necessity for further research. Larger, rigorously controlled trials with longer follow-up periods and systematic assessment of cognitive and functional outcomes are required to refine patient selection criteria, optimize stimulation parameters, and fully delineate the risk-benefit profile of DBS in this challenging clinical population. Furthermore, the foundational neurobiological rationale for targeting specific brain regions continues to evolve alongside advances in functional imaging and circuit neuroscience. Future studies may benefit from the incorporation of biomarkers to predict response, the exploration of novel targets informed by emerging pathophysiological models, and the integration of DBS with adjunctive therapies. In this context, DBS for schizophrenia remains an experimental intervention, but one with the promise to transform the therapeutic landscape for the most refractory cases, contingent upon ongoing scientific and clinical validation.

### 3.5. Treatment-Refractory Addictions

DBS appears effective for treatment-refractory addictions, reducing clinical scores by up to 56% and lowering relapse rates to 8% compared to 85% during early abstinence [75]. Across the current body of literature, the application of DBS in the treatment of intractable addictive disorders has predominantly targeted subcortical regions implicated in reward processing and addiction circuitry, with a particular focus on the ventral striatal area, most notably the nucleus accumbens. This target selection is supported by preclinical evidence suggesting a central role for the nucleus accumbens in the modulation of craving and reinforcement behaviors associated with addictive substances [76]. While the nucleus accumbens was the principal target in the majority of studies, alternative regions such as the bed nucleus of the stria terminalis [77], anterior cingulate cortex [78], and subthalamic nucleus [79] have also been explored. Bilateral implantation of stimulation leads was the standard approach, with only rare exceptions where unilateral placement was employed [80].

The parameters of stimulation applied across these interventions were individualized and adjusted according to clinical response and the emergence of adverse effects. Frequencies ranged from 130 to 185 Hz, with pulse widths spanning 90 to 240 μs. Amplitude settings varied considerably, and individual titration was often necessary to optimize outcomes and minimize side effects [80]. For instance, in one case, anxiety and hypomania observed at 4.5 V were mitigated by reducing the amplitude to 3.7 V, with a further decrease to 3.3 V addressing insomnia and bruxism [81]. While higher voltages were associated with increased efficacy in some cases of tobacco use disorder [82], subsequent investigations found that lower voltages were more suitable for patients with alcohol and heroin dependence [83,84]. The highest reported amplitude was 7 V, but this did not prevent relapse in two patients exposed to such high-voltage stimulation [85]. These findings underscore the necessity for careful and individualized titration of stimulation parameters to balance efficacy and tolerability.

Therapeutic benefits of DBS were widely observed, albeit with significant interindividual variability in the degree of response. Consistent reductions in craving were documented, often representing an early and critical step towards abstinence. For example, even in cases where long-term abstinence was not achieved and tragic outcomes such as fatal overdose occurred, marked reductions in craving were reported in the interim [86]. The majority of patients did not attain complete and sustained abstinence, but many experienced substantial reductions in substance use and episodes of heavy consumption. The translation of these improvements into long-term psychosocial benefits was less frequently documented, though selected reports highlighted positive changes such as employment acquisition, marriage, improved family relationships, and enhancements in social functioning [81,87,88,89,90]. Conversely, some patients experienced negligible benefit, with persistent substance use or substitution with other substances. Notably, in one case, a patient who initially remained abstinent from alcohol for 16 months experienced a series of relapses, which were ultimately attributed to electrode dislocation confirmed on radiographic imaging [88,91]. In other reports, temporary improvement was observed, followed by relapse, sometimes linked to technical factors such as inaccurate electrode placement or device malfunction [81,85,92]. In certain cases, persistent neuropsychiatric comorbidities such as severe depression complicated the clinical course and limited the therapeutic impact of DBS, necessitating additional interventions such as hospitalization for detoxification [93]. Overall, DBS appears to be more effective for alcohol and opioid use disorders than for nicotine use disorder, but limited studies and high heterogeneity prevent firm conclusions [75].

A nuanced spectrum of outcomes following DBS has been described, with abstinence, partial relapse, and full relapse representing key clinical benchmarks. While a proportion of patients achieved sustained abstinence throughout the observation period, a larger subset exhibited intermittent relapses, and a minority experienced complete relapse. The duration of follow-up varied widely, from approximately 100 days to as long as eight years, complicating direct comparisons and interpretation of long-term efficacy. Longer follow-up intervals are particularly valuable in assessing the durability of response and the risk of late relapse, which remains a persistent concern in the management of treatment-refractory addictions [91]. Several factors contributed to relapse, including technical issues such as displaced electrodes [81,88,91], device-related problems (e.g., depleted batteries) [84], and persistent or inadequately treated comorbid psychiatric conditions [86,93]. In some cases, relapse was associated with the development of new patterns of substance use, including the consumption of substances other than the initial target of intervention [82,84,86,93]. These observations highlight the complex interplay between neurological, psychosocial, and environmental factors in determining the ultimate outcome of DBS for treatment-refractory addictions.

Improvements in neuropsychiatric status were frequently observed when abstinence or reduced consumption was achieved. For instance, Bach et al. [94] reported significant reductions in anhedonia and depressive symptoms, with improved quality-of-life in patients receiving active DBS compared to those on sham stimulation. Mean scores for the Snaith-Hamilton Pleasure Scale, Beck Depression Inventory, Hamilton Depression Rating Scale, and Global Assessment of Functioning all favored the DBS group after six months, although statistical significance was not consistently achieved across all measures. Other studies noted substantial reductions in depressive symptoms [78,93], with one reporting a decrease by 63.5% following DBS, though effects on anxiety were less pronounced [78]. Nonetheless, the relationship between neuropsychiatric improvement and substance use outcomes was not always straightforward. Some patients with persistent comorbidities failed to benefit clinically from DBS, even when objective reductions in craving or substance use were noted [86,93]. Conversely, improvements in neuropsychiatric symptoms often accompanied reductions in substance use, suggesting a synergistic effect that supports recovery and enhances overall well-being [95,96]. Reports of weight gain, improved sexual functioning, and enhanced social relationships further underscore the potential for DBS to facilitate holistic recovery in selected patients. However, reporting of quality-of-life outcomes was inconsistent across studies, with most providing only brief or subjective accounts. Only a minority employed formal assessment tools to quantify quality-of-life changes [84,85,94,96], limiting the ability to draw definitive conclusions regarding the broader psychosocial impact of DBS.

Outcomes following DBS were influenced by a constellation of technical and patient-related factors. Accurate targeting and secure placement of electrodes were critical for optimal response, as illustrated by cases where misplaced or dislodged leads resulted in suboptimal outcomes or relapse [81,88,91]. The necessity for individualized programming of stimulation parameters was underscored by reports of side effects such as insomnia, hypomania, and bruxism, which often resolved with parameter adjustment [81,85]. Device-related complications, such as battery depletion, also contributed to relapse in some cases [84].

Patient motivation, psychosocial context, and the presence of comorbid psychiatric disorders emerged as key determinants of therapeutic success. Lack of motivation, persistent neuropsychiatric symptoms, and environmental factors such as exposure to substance-using peers or settings were frequently cited as barriers to sustained abstinence and recovery [82,84,86,90,93]. A tendency toward poly-substance abuse was also observed in some individuals, often linked to “boredom” or habitual behaviors, further complicating the clinical picture [82,84,86,93].

With all this, the evidence base for DBS in treatment-refractory addictions is constrained by several limitations. Small sample sizes, lack of randomization and blinding in most studies, and reliance on self-reported outcomes introduce the potential for bias and limit generalizability. Heterogeneity in outcome measures, variable follow-up durations, and inconsistent reporting of key variables such as quality-of-life and comorbid psychiatric outcomes further complicate interpretation [77,78,79,81,82,83,86,87,88,89,90,91,92,93,94,95,97,98,99,100,101,102,103]. Despite these challenges, the available data suggest that DBS targeting the nucleus accumbens and related structures can yield substantial reductions in craving and substance use for a subset of individuals with refractory treatment-refractory addictions. Therapeutic benefits are most pronounced when technical precision, individualized programming, and comprehensive management of comorbidities and psychosocial factors are achieved.

In summary, DBS represents a promising—yet complex and resource-intensive—therapeutic alternative for treatment-refractory addictions. Its efficacy is modulated by technical, neuropsychiatric, and environmental variables, necessitating a multidisciplinary approach to patient selection, perioperative care, and long-term follow-up. Future research should prioritize rigorous study designs, standardized outcome measures, and longer-term follow-up to better elucidate the role of DBS in the management of treatment-refractory addictions and to optimize patient outcomes.

### 3.6. Intractable Tourette’s Syndrome

Employing standardized outcome measures such as the Yale Global Tic Severity Scale, the available literature indicates that more than two-thirds of patients with severe treatment-refractory Tourette’s syndrome experience a symptom reduction of over 50% following DBS intervention, with a median follow-up period of 12 months. This effect is evident in both RCTs and when non-randomized studies are considered, indicating a robust association between DBS and tic symptom improvement [104,105]. Importantly, analyses of sham DBS treatments do not show significant changes in Yale Global Tic Severity Scale, thereby strengthening the attribution of observed improvements to the active stimulation itself rather than to surgical intervention or placebo effects [104].

Subgroup analyses have investigated how the placement of DBS electrodes affects therapeutic outcomes in patients with tics. Evidence suggests that stimulating the thalamic ventrooral region leads to greater reductions in Yale Global Tic Severity Scale scores compared to targeting the globus pallidus internus, indicating that anatomical target selection may influence clinical efficacy. However, this conclusion should be interpreted cautiously due to the limited number of studies examining the ventrooral thalamus [104]. Other analyses show that thalamic DBS produces lower tic reduction rates than pallidal DBS during the first 12 months after surgery. However, this benefit seems to be confined to the first three months postoperatively. Importantly, the current body of evidence, including recent findings, suggests that the full therapeutic benefits of DBS for tic reduction often require at least one year to become evident. Specifically, Yale Global Tic Severity Scale scores show more substantial improvement at least one year after DBS of the thalamic centromedian-parafascicular nucleus compared to earlier post-surgical evaluations, highlighting the critical role of long-term follow-up in assessing DBS efficacy. These observations emphasize the necessity for future research to include extended observation periods in order to accurately determine the long-term effectiveness and comparative efficacy of different DBS targets in tic disorder treatment [106,107,108,109,110,111]. Overall, there is no consistent evidence of significant differences in tic reduction between different DBS targets [106]; for example, some studies report tic improvements of 97% with subthalamic nucleus stimulation [112], 70.5% with external globus pallidus stimulation [113], and 44% with internal capsule/nucleus accumbens stimulation [107].

The effectiveness of DBS depends on carefully adjusting stimulation parameters, which include frequency (commonly set around 130 Hz), pulse width (usually low, about 60–120 μs), and amplitude (typically between 2 and 5 volts) [114,115]. These parameters are not fixed and must be tailored to each individual patient. Clinicians adjust the amplitude, pulse width, and frequency based on the patient’s unique response and the particular brain region being targeted. This individualized approach helps to maximize symptom relief while minimizing potential side effects, as the optimal DBS settings can vary considerably from one patient to another [105].

Adverse effects of DBS can also be categorized as either procedure-related or stimulation-related, with some stimulation effects varying by target region. For example, stimulation of the thalamic centromedian-parafascicular region is linked to gaze disturbances and visual symptoms, while these are less common with motor thalamic targets [116]. Modulation of intralaminar thalamic nuclei may also affect sexual function, leading to increased or decreased libido [117]. Other reported effects include arm paresthesia, dysarthria, and rare psychiatric symptoms such as psychosis. Stimulation of the posteroventral globus pallidus internus has been associated with increased anxiety, depression, and memory impairment [118]. In contrast, stimulation of the anteromedial globus pallidus internus has been linked to higher anxiety, dyskinetic limb movements, and a case of hypomania [119]. Targeting the anterior limb of the internal capsule/nucleus accumbens can lead to depression, hypomania, and, in one instance, a suicide attempt in a patient with a pre-existing depressive disorder. More common adverse effects across all targets include apathy, fatigue, dizziness, and weight changes, which can often be mitigated by adjusting stimulation parameters. However, careful target selection is essential to minimize these side effects. Procedure-related complications primarily involve hardware malfunction and infections. Infection rates are notably higher in Tourette’s syndrome patients (18%) compared to other DBS indications (overall 3.7%) [120], possibly due to compulsive picking at surgical scars or altered immune function such as lower T cell counts, dysgammaglobulinemia, or dopamine-mediated immunomodulation [120,121,122]. Further research is needed to clarify these mechanisms.

Several methodological limitations are also evident across the current evidence base. The diversity of patient populations—in terms of age, comorbidities, and concurrent medication use—may contribute to variability in treatment responsiveness. The limited number of high-quality RCTs and the potential for publication bias, due to underreporting of negative or inconclusive results, further restrict the strength of the available conclusions.

In aggregate, the current literature provides strong evidence that DBS significantly reduces tic severity in patients with intractable Tourette’s syndrome, outperforming sham interventions and demonstrating a clear superiority over repetitive transcranial magnetic stimulation in RCT-based analyses. The magnitude of benefit may be influenced by the anatomical site of electrode implantation, with thalamic ventrooral stimulation showing the greatest promise. Despite these encouraging results, the risk of adverse effects and the need for tailoring anatomical targeting highlight the complexity of DBS as a therapeutic modality. The field would benefit from direct comparative trials, standardization of stimulation and outcome protocols, and comprehensive safety reporting to further delineate the optimal role of DBS in the management of severe treatment-refractory Tourette’s syndrome. Addressing these research gaps will be critical for refining patient selection, maximizing therapeutic benefit, and minimizing risk in this challenging neuropsychiatric disorder.

### 3.7. Treatment-Refractory Anorexia Nervosa

DBS has emerged as a promising intervention for treatment-refractory anorexia nervosa, a severe psychiatric disorder characterized by persistent restriction of energy intake and an intense fear of gaining weight, often resistant to conventional treatment modalities. Evidence from recent clinical investigations has begun to elucidate the potential benefits and safety profile of DBS in this challenging patient population. DBS targets varied among studies, with the nucleus accumbens, subcallosal cingulate cortex, and ventral anterior limb of the internal capsule being the most commonly investigated sites. Target selection was at times individualized, particularly based on predominant psychiatric comorbidities. Across studies, a minority of patients required early electrode explantation due to complications or lack of efficacy [123,124,125].

The most robust finding across these investigations was a significant and clinically meaningful increase in body mass index (BMI) following DBS [126,127,128,129,130,131,132]. Meta-analytic synthesis demonstrated a large effect size for BMI improvement, indicating that DBS may exert a substantial impact on weight restoration in patients otherwise unresponsive to standard therapies. This effect persisted across studies targeting different brain regions and across varying lengths of follow-up, ranging from six months to two years [123,124,125]. While the specific magnitude of BMI changes varied, the consistent directionality suggests a therapeutic benefit of DBS in ameliorating the core symptomatology of anorexia nervosa [123]. In addition to BMI, secondary outcome measures included assessments of psychiatric comorbidity, quality of life, and global functioning. Improvements were frequently reported in depressive and obsessive-compulsive symptoms, as well as in measures of anxiety and overall psychological wellbeing. Notably, these gains were observed without major changes to psychotropic medication regimens, supporting the hypothesis that DBS may directly modulate neural circuits implicated in both anorexia nervosa and its common psychiatric comorbidities [127,129]. Quality of life and social functioning also trended towards improvement, although the degree of change varied among individuals and studies [123].

Safety and tolerability of DBS in this population were generally favorable, despite the inherent procedural risks of neurosurgery. Adverse events were relatively infrequent and predominantly mild to moderate in severity, including transient mood alterations, wound infections, and device-related discomfort. Only a small number of patients required device removal before completion of follow-up. There was no evidence of worsening psychiatric symptoms or emergence of new psychopathology directly attributable to DBS [126,128,129]. Nonetheless, the surgical intervention remains invasive, and careful patient selection and monitoring are imperative [123].

Importantly, the non-randomized, non-controlled design of the available studies limits the ability to draw firm causal inferences regarding efficacy. The patient cohorts were highly selected, and placebo effects or regression to the mean cannot be ruled out. Furthermore, the heterogeneity in DBS targets and stimulation parameters complicates direct comparison across studies and precludes definitive conclusions regarding optimal neuroanatomical sites for intervention. Despite these limitations, the accumulating clinical data indicate that DBS may offer a viable treatment strategy for individuals with chronic, severe, and otherwise intractable anorexia nervosa. The observed improvements in BMI and psychiatric comorbidity are encouraging, particularly in a population at high risk for medical morbidity and mortality. Further research, ideally employing randomized controlled designs and larger, more diverse samples, is needed to confirm these preliminary findings, elucidate mechanisms of action, and refine patient selection criteria [126,127,128,129]. In summary, while DBS remains an experimental modality for treatment-refractory anorexia nervosa, its application in carefully selected patients is associated with significant clinical improvement, reinforcing the role of neuromodulation in the evolving landscape of anorexia nervosa therapeutics.

### 3.8. Treatment-Refractory Post-Traumatic Stress Disorder

The application of DBS in treatment-refractory post-traumatic stress disorder represents a novel intervention for individuals unresponsive to conventional pharmacological and psychotherapeutic approaches. The selection of DBS targets in post-traumatic stress disorder has been informed both by preclinical animal models and by human neuroimaging studies, particularly those highlighting hyperactivity in specific brain regions such as the basolateral amygdala during trauma-related recall [133]. The basolateral amygdala, implicated in pathological fear processing, has thus emerged as a plausible target for modulation [134]. A pilot clinical investigation targeting the basolateral amygdala has yielded detailed longitudinal outcomes for two patients with chronic, severe post-traumatic stress disorder. In the first reported case, a combat veteran suffering from persistent symptoms for two decades despite exhaustive standard interventions underwent bilateral basolateral amygdala DBS, with implantation guided by advanced imaging and stereotactic techniques [135,136,137,138]. Baseline post-traumatic stress disorder severity, as measured by the Clinician-Administered Post-Traumatic Stress Disorder Scale (CAPS), was categorized as extremely severe (score of 119). Neuroimaging during trauma recall confirmed hypermetabolism in the amygdala relative to resting state, supporting the rationale for basolateral amygdala targeting.

Surgical implantation was executed using a trajectory designed to traverse the central nucleus of the amygdala, the basolateral amygdala, and the head of the hippocampus, with careful avoidance of critical vascular and cisternal structures [136]. Following DBS initiation, the patient demonstrated marked clinical improvement: at 8 months postoperatively, the CAPS score had declined to 74, representing a 37.8% reduction, and further dropped to 62 (a 48% reduction) at 15 months [135,137]. Stimulation was applied at 130 Hz with a pulse width of 60 μs and an individualized amplitude range of 2.0–6.5 mA for each hemisphere [139]. Notably, the patient experienced over a year of near-complete suppression of severe nightmares, a hallmark post-traumatic stress disorder symptom [138]. Nevertheless, the course was not without complications, as the patient required hospitalization for suicidality at 17 months post-DBS. Despite this, at a 4-year follow-up, the patient maintained a 40% reduction in post-traumatic stress disorder symptom severity [138].

A second patient, also a combat veteran, underwent basolateral amygdala DBS and exhibited over 30% improvement in CAPS scores within 7 months post-surgery [138,140]. These findings are especially noteworthy, as sustained symptom reduction in such treatment-resistant cases is uncommon with existing therapies. Importantly, these cases provide some of the longest follow-up data available on DBS for post-traumatic stress disorder, suggesting both the feasibility and potential durability of this intervention.

Another exploratory approach has targeted the medial prefrontal cortex and its white matter connections, specifically the uncinate fasciculus, based on preclinical evidence implicating these structures in fear extinction and emotional regulation [141,142]. In this case, a patient with a long-standing history of post-traumatic stress disorder secondary to domestic abuse underwent DBS with leads centered on the subgenual cingulum, maximizing contact with the uncinate fasciculus and adjacent fiber tracts [141]. This targeting strategy was facilitated by multimodal MRI and diffusion tensor imaging to precisely localize relevant white matter bundles.

The clinical outcome was striking: at 6 months following surgery, the patient’s CAPS score had improved by 100%, signifying complete remission of post-traumatic stress disorder symptoms. This was accompanied by marked enhancements in mood, global functioning, and quality of life [141]. The specific contribution of stimulating the uncinate fasciculus and related frontolimbic circuits remains to be elucidated, but these results underscore the potential of targeting frontoamygdalar pathways in refractory post-traumatic stress disorder [134].

Taken together, the available narrative evidence suggests that DBS, particularly when directed at the basolateral amygdala or medial prefrontal cortex/uncinate fasciculus regions, can induce substantial and durable improvements in core post-traumatic stress disorder symptoms in patients unresponsive to standard treatments. The observed benefits include not only overall reductions in post-traumatic stress disorder severity but also pronounced amelioration of specific symptoms such as nightmares and functional impairment. These clinical outcomes were corroborated by objective neuroimaging findings, supporting the neurobiological rationale for these targets. Nevertheless, the narrative review of these cases highlights several caveats. The small number of reported cases and the complexity of patient histories preclude definitive conclusions regarding efficacy, generalizability, or optimal stimulation parameters. Furthermore, while therapeutic response appears robust in the context of severe, chronic post-traumatic stress disorder, potential adverse effects, such as the emergence of suicidality, warrant careful monitoring and multidisciplinary management. Surgical targeting, electrode placement strategies, and stimulation protocols remain heterogeneous, reflecting the nascent stage of research in this area.

In summary, DBS targeting the basolateral amygdala and medial prefrontal cortex/uncinate fasciculus demonstrates promising results in highly selected, treatment-refractory post-traumatic stress disorder cases, achieving clinically meaningful symptom reductions and enhanced quality of life over extended follow-up periods [135,136,137,138,139,140,141]. While these findings provide compelling preliminary support for the utility of DBS in refractory post-traumatic stress disorder, further research involving larger patient cohorts and controlled designs is critical to establish efficacy, optimize targeting strategies, and clarify the risk-benefit profile of this invasive intervention [143].

### 3.9. Refractory Aggression in Autistic Children with Severe Intellectual Disability

DBS is used as a neurosurgical treatment for treatment-resistant aggression in autistic children with severe intellectual disability, especially when standard therapies fail [144]. The primary clinical indication for DBS remains the mitigation of uncontrolled aggression, although movement disorders and excessive agitation have also justified its use [5,6,12,23,24,25,30,31,32,33,34,36,37,38,39,40,41,145,146,147,148,149,150,151,152,153,154,155,156,157,158,159,160,161]. The mean follow-up duration in these studies is approximately 42.5 months, with most patients followed for at least one year, allowing for longitudinal assessment of behavioral changes. Notably, one study reported a follow-up period extending to 163 months, reflecting the potential for long-term observation [146,161].

Surgical planning was uniformly conducted using stereotactic techniques, incorporating pre- and postoperative neuroimaging for precise electrode placement. The posteromedial hypothalamic nuclei was the most frequently targeted region [146,147,148,149,150,152,153,161], although alternative targets, including earlier and ventral brain regions, or short-subcortical areas, were explored by some groups [145,151,155,156,157,158,159,160,162]. The technical aspects of electrode implantation and device model were variably reported; the Medtronic 3389 model was most commonly used, but not universally documented. Stimulation parameters—voltage (0.5–6.5 V), frequency (15–185 Hz), and pulse width (60–360 μs)—were inconsistently reported, further highlighting variability in clinical practice [144].

Assessment of treatment efficacy was complicated by the heterogeneous use of outcome measures. Only a minority of studies employed standardized tools to objectively evaluate intellectual disability [145,147,152] or the severity of autism [148,151,156], owing largely to the profound impairments present in these populations. For aggression assessment, the Overt Aggression Scale (OAS) [147,148,149,150,152] and its modified version (MOAS) [145,153,154,155,157,160] were the most commonly used instruments, but several reports either did not employ these scales or did not provide quantitative data [155,157,160,162]. The majority of patients (53/65) were evaluated with objective aggression scales pre- and post-operatively, but clinically meaningful changes were reported in only 51 cases, with a minimum follow-up of 12 months [144]. A remarkable clinical improvement was observed in 94.2% of these patients (48/51), as documented by reductions in aggressive behaviors following DBS. Effect size calculations, although limited to a subset of studies with available data, indicated robust efficacy: OAS-based studies reported effect sizes ranging from 3.71 to 4.81 [148,149,152], and a significant effect size (d = 1.01) was reported in one MOAS-based study. Pooled intra-group analysis across studies demonstrated large effect sizes for both OAS (d = 4.32) and MOAS (d = 1.46), supporting the substantial impact of DBS on aggression reduction [144]. Nevertheless, adverse outcomes were documented. Surgical complications included basal ganglia hemorrhage [149,163] and infections at the operative or device sites, occasionally necessitating device removal [153,155,157]. Some families discontinued treatment due to adverse effects [146,153,161], and adjustments in stimulation parameters were sometimes required to optimize efficacy or mitigate side effects [154,155]. Device-related complications, such as battery depletion, led to relapse of aggressive behaviors, which were generally reversible upon battery replacement [151,153,154,157]. In a minority of cases, the return of aggression or non-adherence to follow-up limited the ability to sustain or assess response [150,158,159].

Overall, despite variability in methodology, target selection, and outcome assessment, the available evidence consistently indicates that DBS offers significant and durable reduction in refractory aggressive behaviors in autistic children with severe intellectual disability. The magnitude of clinical improvement is substantial, with benefits maintained over extended follow-up periods in most cases. However, the procedure is not without risk; surgical and device-related complications, as well as the need for ongoing management and follow-up, must be carefully considered. The current literature supports the potential of DBS as a viable therapeutic option for this challenging population, while emphasizing the need for standardized protocols, rigorous assessment tools, and long-term safety monitoring to optimize outcomes and further delineate its therapeutic profile [144].

## 4. Discussion

The clinical application of DBS across a spectrum of treatment-resistant psychiatric disorders marks a significant advance in neuropsychiatry, offering hope where conventional modalities have failed. Accumulating evidence from diverse diagnostic entities—including major depressive disorder, obsessive–compulsive disorder, bipolar disorder, schizophrenia, addiction, Tourette’s syndrome, anorexia nervosa, post-traumatic stress disorder, and severe aggression in autistic children with intellectual disability—demonstrates the potential of DBS to modulate dysfunctional neural circuits and achieve symptom reduction in otherwise intractable cases. However, this promise is tempered by methodological, ethical, and practical complexities that must inform interpretation and guide next steps in both research and clinical translation.

A core challenge lies in the considerable heterogeneity that characterizes the extant literature. Across disorders, studies vary in design, sample size, target selection, and stimulation protocols. Most evidence derives from open-label, single-arm, or small cohort studies, with RCTs remaining the exception rather than the rule. This is particularly salient in disorders such as bipolar disorder, schizophrenia, and anorexia nervosa, where the data base remains largely limited to case reports and small series [56,57,58,65,66,67,68,69,126,127,128,129]. Even in conditions with more substantial evidence—such as treatment-resistant depression and obsessive–compulsive disorder—RCTs often suffer from small sample sizes, high attrition rates, and short sham-controlled periods [18,21,22,23,30,31,32,33,34,35,36,37,38,39,40,41,42,43,44,45,46,47,48,49,50,51,104,105]. These design limitations restrict the generalizability of findings and amplify the risk of type I and II errors.

An additional methodological limitation is the inconsistent reporting of patient-level data and the lack of standardized outcome measures, particularly regarding quality of life and functional recovery. Many studies focus narrowly on symptom reduction using disorder-specific scales but neglect broader indices of psychosocial functioning, cognitive impact, and long-term wellbeing [54,84,94,96]. This constrains the ability to fully evaluate the real-world benefit of DBS and to compare outcomes across diagnostic boundaries. Moreover, the high degree of interindividual variability in response—evident in all conditions reviewed—underscores the need for granular analyses that can elucidate predictors and moderators of treatment effect.

The identification of optimal DBS targets remains an area of active investigation and uncertainty. While some targets, such as the subcallosal cingulate in depression or the ventral capsule/ventral striatum in obsessive–compulsive disorder, have shown promise, head-to-head comparisons are rare, and superiority of one anatomical site over another is seldom statistically established [14,15,16,30,31,32,33,34,35,36,37,38,39,40,41,42,43,44,45,46,49,50,51,104]. The choice of target is often based on pathophysiological models and neuroimaging findings but is complicated by the evolving understanding of neural circuit dysfunction in psychiatric illness. Furthermore, the necessity for individualized selection based on comorbidities or predominant symptom clusters—as seen in anorexia nervosa and post-traumatic stress disorder—adds another layer of complexity [123,124,125,134,135,136,137,138,141].

Stimulation parameters, too, lack standardization. Most protocols employ high frequencies and relatively uniform pulse widths, yet amplitude and lead configuration are frequently titrated empirically to patient response [17,53,80,114,115]. This individualized approach, while clinically pragmatic, complicates the synthesis and comparison of results. Importantly, no robust relationship between specific stimulation parameters and clinical response has been established, suggesting that factors beyond the technical settings—such as neuroanatomical precision, disease chronicity, and patient motivation—play a substantial role in determining outcomes [17,81,85].

The aggregate findings across disorders and targets suggest that DBS does not act via a single universal mechanism but rather through modulation of network activity that may involve both excitation and inhibition, depending on the site and pattern of stimulation. For example, stimulation of the subcallosal cingulate in depression is hypothesized to inhibit hyperactive limbic circuits, restoring a balance between affective and cognitive control regions [14,15,16]. In obsessive–compulsive disorder, targeting the ventral capsule/ventral striatum may reduce pathological connectivity within the cortico-striato-thalamo-cortical loop, again suggesting a mechanism of rebalancing dysfunctional network dynamics [30,31,32,33,34,35,36,37,38,39,40,41,42,43,44,45,46]. While most protocols employ high-frequency stimulation, which is traditionally associated with local inhibition, emerging evidence also points to complex downstream effects—potentially including disinhibition of target areas and facilitation of adaptive connectivity [17,81,85]. Thus, rather than simply suppressing or activating a given structure, DBS appears to restore homeostasis within pathological circuits by modulating abnormal patterns of activity and connectivity. Future studies leveraging advanced neuroimaging and electrophysiology will be crucial to delineate whether certain parameter/target combinations preferentially induce excitation, inhibition, or network-level rebalancing in specific disorders.

Across all disorders, the safety profile of DBS is generally favorable, with most adverse events relating to device- or procedure-related complications, such as infections, lead displacement, or hardware malfunction [14,31,45,149,163]. Serious neurological sequelae, including hemorrhage or seizure, are rare but underscore the invasive nature of the intervention. Psychiatric adverse events—such as mood switches in bipolar disorder or emergence of obsessive-compulsive symptoms focused on the device—highlight the need for ongoing psychiatric monitoring and parameter adjustment [45,56,60,118,119]. Device-related complications, such as battery depletion, may precipitate symptom relapse but are typically reversible upon intervention [14,84,151,153,154,157]. Notably, the risk of suicide in severely ill populations undergoing DBS appears comparable to that seen in other neuromodulation cohorts, without clear evidence of direct causality [26,27,28,29]. Nonetheless, these risks must be carefully balanced against the morbidity and mortality associated with the underlying illness, particularly in disorders such as anorexia nervosa and severe aggression in children with autism, where conventional treatments have failed and prognosis is poor [123,124,125,126,127,128,129,144].

The durability of DBS response is a critical consideration. Data from depression, obsessive–compulsive disorder, Tourette’s syndrome, and addiction reveal that clinical improvements are typically maintained over intermediate to long-term follow-up, with the most pronounced gains observed in the first 6–12 months post-implantation and a subsequent plateau [14,15,16,17,18,19,91,104,105]. However, relapse can occur, often in association with technical failures or environmental stressors [88,91,151,153,154,157]. In schizophrenia and post-traumatic stress disorder, symptom exacerbation reliably follows cessation of stimulation, underscoring the direct therapeutic effect of DBS [69,138]. These findings emphasize the need for lifelong follow-up and the establishment of multidisciplinary care models that can address both device management and psychiatric comorbidity.

The clinical implications of DBS in psychiatry are profound. For individuals with severe, chronic, and otherwise intractable illness, DBS offers a potential avenue for symptom relief and functional recovery. In depression and obsessive–compulsive disorder, response and remission rates approach or exceed those of other neuromodulatory interventions, and the magnitude of improvement often represents a transformative change in quality of life [14,15,16,17,18,19,30,31,32,33,34,35,36,37,38,39,40,41,42,43,44,45,46,49,50,51]. Clinical application priorities should focus on patients with treatment-resistant depression who have not responded to at least two antidepressants plus psychotherapy, and who do not have serious organic diseases or uncontrolled medical conditions. For OCD, DBS is prioritized for those with severe, persistent symptoms unresponsive to optimal pharmacological and behavioral therapy. The extension of DBS to other domains—such as bipolar depression, schizophrenia, and addiction—remains experimental but is supported by preliminary evidence of efficacy and safety in highly selected populations [56,57,58,65,66,67,68,69,75,76,77,78,79,80,81,82,83,84,85,86,87,88,89,90,91,92,93,94,95,96,97,98,99,100,101,102,103]. Core criteria for patient selection in these disorders include documented failure of standard treatments, chronicity and severity of symptoms, and careful exclusion of patients with unstable medical or psychiatric conditions. For example, patients with schizophrenia considered for DBS should not be in an acute psychotic episode, and individuals with autism-related aggressive behavior should not be in an acute psychiatric crisis at the time of intervention. In pediatric populations with severe aggression, DBS not only reduces the burden of violence but may also improve overall adaptive functioning, offering relief to caregivers and reducing the need for institutionalization [144,145,146,147,148,149,150,151,152,153,154,155,156,157,158,159,160,161,162]. However, patient selection in pediatric cases should exclude those exhibiting aggression during acute psychotic episodes, and clinicians should prioritize cases where standard interventions have failed, and aggression substantially impairs function or safety.

Despite these promising findings, several caveats must temper clinical enthusiasm. DBS is not curative and does not benefit all patients; nonresponse, partial response, and relapse remain common. The invasive, resource-intensive nature of the procedure restricts its application to the most severely affected individuals, and robust criteria for patient selection—including psychiatric stability, motivation, and absence of contraindications—are paramount. The potential for neuropsychiatric adverse effects, while generally manageable, requires structured monitoring and rapid intervention. Moreover, the ethical considerations surrounding irreversible neurosurgical procedures in vulnerable populations, particularly children and those with impaired decision-making capacity, demand rigorous oversight and informed consent.

Future directions in DBS research and clinical practice should prioritize several key areas. First, adequately powered RCTs with longer sham-controlled phases, standardized stimulation and outcome protocols, and comprehensive reporting of functional and quality-of-life outcomes are necessary to refine efficacy estimates and facilitate meta-analytic synthesis [18,104,105,126,127,128,129]. Second, advances in neuroimaging, connectomics, and computational modeling—particularly the application of patient-specific computational models to predict optimal stimulation parameters and target selection—should inform more precise targeting of pathological circuits and individualized parameter optimization. The integration of biomarkers—whether neurophysiological, neuroimaging, or genetic—may enable prediction of responders and personalization of therapy. Moreover, closed-loop DBS systems, which dynamically adjust stimulation in response to real-time neural signals, represent a promising paradigm shift toward adaptive and personalized neuromodulation in psychiatry. Third, the exploration of novel targets, including white matter tracts and frontoamygdalar circuits, should be pursued in a hypothesis-driven, ethically guided manner [134,135,136,137,138,139,140,141,142]. Fourth, the long-term cognitive, psychosocial, and neurodevelopmental impact of DBS, especially in pediatric and young adult populations, warrants systematic study [60,123,124,125,144,145,146,147,148,149,150,151,152,153,154,155,156,157,158,159,160,161,162]. Interdisciplinary collaboration will be essential—encompassing psychiatry, neurosurgery, neurology, neuropsychology, and ethics—to optimize patient selection, perioperative care, and follow-up. The development of consensus guidelines and registries will facilitate data sharing, safety monitoring, and the refinement of best practices. Finally, patient and caregiver perspectives must be integrated into both research and clinical decision-making, ensuring that the ultimate aim of DBS—to restore agency, autonomy, and quality of life—is realized.

## 5. Conclusions

In conclusion, DBS represents a transformative but complex intervention in the management of treatment-resistant psychiatric disorders. While robust evidence supports its efficacy in select populations, limitations in study design, heterogeneity of practice, and the need for individualized approaches must be acknowledged. The balance between benefit and risk will continue to evolve alongside advances in neuroscience, clinical methodology, and ethical frameworks. Ongoing research, guided by scientific rigor and clinical humility, will determine the ultimate place of DBS in the therapeutic armamentarium for refractory psychiatric illness.

## Figures and Tables

**Table 1:** Comparative table of DBS efficacy for different treatment-resistant psychiatric disorders.

Disorder	Main Target (s)	Response Rate (%)	Remission Rate (%)	Mean Follow-Up Duration	Main Outcome Measure(s)
TRMDD	mFB, VC/VS, NAc, SCG, BNST, etc.	~48%	~35%	21 months	MADRS, HDRS
TROCD	VC/VS, NAc, ALIC, STN, BNST	~66%	N/A	Up to 24 months	Y-BOCS
TRBD	VC/VS, mFB, SCG	~48%	N/A	Up to 9 months	MADRS, HDRS
TRS	sgACC, NAc, habenula, SNr	Variable (~40%)	N/A	Up to 36 months	PANSS, BPRS
TRA	NAc, BNST, ACC, STN	Up to 56% (craving)	N/A	Up to 8 years	Craving Scores, Relapse Rate
ITS	CM-Pf, GPi, NAc, Vo thalamus	>66% (tic reduction > 50%)	N/A	12 months (median)	YGTSS
TRAN	NAc, SCG, vALIC	Significant BMI increase	N/A	6–24 months	BMI, QOL, Psychiatric Scales
TRPTSD	BLA, mPFC/uncinate fasciculus	>30–100% (case reports)	N/A	Up to 4 years	CAPS
RAAID	PMH, other hypothalamic targets	>90% (aggression reduction)	N/A	42.5 months (mean)	OAS, MOAS

Note. TRMDD—treatment-resistant major depressive disorder; TROCD—treatment-resistant obsessive–compulsive disorder; TRBD—treatment-resistant bipolar disorder; TRS—treatment-resistant schizophrenia; TRA—treatment-refractory addictions; ITS—intractable Tourette’s syndrome; TRAN—treatment-refractory anorexia nervosa; TRPTSD—treatment-refractory post-traumatic stress disorder; RAAID—refractory aggression in autistic children with severe intellectual disability; mFB—medial forebrain bundle; VC/VS—ventral capsule/ventral striatum; NAc—nucleus accumbens; SCG—subgenual cingulate gyrus/cortex; BNST—bed nucleus of the stria terminalis; ALIC—anterior limb of internal capsule; STN—subthalamic nucleus; sgACC—subgenual anterior cingulate cortex; SNr—substantia nigra pars reticulata; ACC—anterior cingulate cortex; CM-Pf—centromedian-parafascicular thalamic nucleus; GPi—globus pallidus internus; Vo thalamus—ventrooral thalamus; vALIC—ventral anterior limb of internal capsule; BLA—basolateral amygdala; mPFC—medial prefrontal cortex; PMH—posteromedial hypothalamus; MADRS—Montgomery–Åsberg Depression Rating Scale; HDRS—Hamilton Depression Rating Scale; QOL—quality of life; OAS—Overt Aggression Scale; MOAS—Modified Overt Aggression Scale; CAPS—Clinician-Administered PTSD Scale; PANSS—Positive and Negative Syndrome Scale; BPRS—Brief Psychiatric Rating Scale; Y-BOCS—Yale-Brown Obsessive Compulsive Scale; YGTSS—Yale Global Tic Severity Scale; N/A—not available or not reported; BMI—Body Mass Index.

**Table 2:** Statistical chart of common DBS SE(s).

Disease/Indication	Device-Related SE(s)	Neurological SE(s)	Psychiatric SE(s)	Other/Procedure-Related SE(s)	Notable Features
TRMDD	~7–10% (infection/device), battery failure < 5%	Seizure: rare; hemorrhage: very rare	Suicidal ideation: ~1% per 100 person-years	Wound issues	No increased suicide risk
TROCD	5–10% (infection, lead damage/explant)	Seizure: rare; hemorrhage: <2%	New OCD symptoms (rare), suicidality	Dysarthria, paresis	Device can become OCD focus
TRBD	Sparse data	Not systematically reported	Hypomania/mania (case reports; reversible)	–	Risk of affective switching
TRS	Device infection, hemorrhage (2 cases)	Seizure, confusion, akathisia	Mood lability (esp. withdrawal of meds)	Weight change	Most AEs reversible
TRA	Infection, battery depletion	Seizure (very rare), insomnia	Hypomania, anxiety, depression	Device malfunction	SEs often parameter-dependent
ITS	Infection: ~18% (higher than other DBS)	Paresthesia, dysarthria, gaze/visual disturbance	Anxiety, depression, memory changes	Hardware malfunction	Compulsive wound picking risk
TRAN	| Infection, device discomfort (rare explant)	Transient mood change, wound problems	No major psychiatric worsening	–	No new psychopathology
TRPTSD	Not specified	Not specified	Suicidality (case report)	–	Longest follow-up: 4 years
RAAID	Infection, device failure, hemorrhage	Hemorrhage (rare), not systematically reported	Not specified (communication limited)	Device removal	Device-related relapse

Note. DBS—deep brain stimulation; SE(s)—side effect(s); TRMDD—treatment-resistant major depressive disorder; TROCD—treatment-resistant obsessive–compulsive disorder; TRBD—treatment-resistant bipolar disorder; TRS—treatment-resistant schizophrenia; TRA—treatment-refractory addictions; ITS—intractable Tourette’s syndrome; TRAN—treatment-refractory anorexia nervosa; TRPTSD—treatment-refractory post-traumatic stress disorder; RAAID—refractory aggression in autistic children with severe intellectual disability.

## Data Availability

No new data were created or analyzed in this study. Data sharing is not applicable to this article.
